# The Causes and Role of Antinatalism in Poland in the Context of Climate Change, Obstetric Care, and Mental Health

**DOI:** 10.3390/ijerph192013575

**Published:** 2022-10-20

**Authors:** Filip Franciszek Karuga, Bartosz Szmyd, Karolina Petroniec, Aleksandra Walter, Agnieszka Pawełczyk, Marcin Sochal, Piotr Białasiewicz, Dominik Strzelecki, Maria Respondek-Liberska, Monika Tadros-Zins, Agata Gabryelska

**Affiliations:** 1 Department of Sleep Medicine and Metabolic Disorders, Medical University of Lodz, 90-419 Lodz, Poland; 2 Department of Neurosurgery, Spine and Peripheral Nerves Surgery, Medical University of Lodz, 90-549 Lodz, Poland; 3 Department of Pediatrics, Oncology, and Hematology, Medical University of Lodz, 91-738 Lodz, Poland; 4 Specialist Regional Hospital in Ciechanow, 06-400 Ciechanow, Poland; 5 Pabianice Medical Center Sp. o.o., 95-200 Pabianice, Poland; 6 Department of Affective and Psychotic Disorders, Medical University of Lodz, 92-215 Lodz, Poland; 7 Department for Diagnoses and Prevention, Medical University of Lodz, 93-338 Lodz, Poland; 8 Department of Prenatal Cardiology, Polish Mother’s Memorial Hospital, 93-338 Lodz, Poland; 9 Department of Obstetrics and Gynecology, Polish Mother’s Memorial Hospital, 93-338 Lodz, Poland

**Keywords:** environmental antinatalism, climate, environmentalism, depression, anxiety, childless

## Abstract

Antinatalism is an umbrella term for numerous moral dilemmas associated with procreation. In the past few years, the deterioration of environmental conditions, social difficulties, global worsening of people’s mental health, and pandemics have induced discussion about antinatalism. Therefore, we aimed to characterize antinatalists in the Polish population in terms of the frequency and description of the main reasons behind this phenomenon. The cross-sectional study was performed in the Polish population. An online, four-part survey was performed between 19 and 25 January 2022. The study group comprised 1240 respondents. Antinatalists (*n* = 472, 38%) were defined as people who do not have children and want to be childless in the future, whereas pronatalists (*n* = 768, 62%) consisted of people who want to have offspring in the future and/or already have children. The opinion that climate change is a significant reason not to have a child appeared twice as often among antinatalists. Additionally, the performed binary logistic regression model highlighted the importance of the fear of climate change as an independent factor facilitating an antinatalistic attitude. Regarding females, the following factors discouraging them from having a child were observed: fear of child’s congenital diseases, pregnancy complications, dissatisfaction with medical services, and fear of exacerbation of maternal chronic diseases. Anxiety, depression, and stress were not found to be statistically different between pro- and antinatalist groups. However, further analysis revealed that female antinatalists were significantly more depressive and anxious. Our study helps us to understand why, as mentioned beforehand, around 38% of respondents prefer to stay childless. In conclusion, antinatalism views have become relatively prevalent in society, and its reasons include environmental antinatalism and medical factors, including depression and anxiety. However, better access to medical services and changes in climate politics were not found to be significant factors in encouraging society to decide to have offspring.

## 1. Introduction

Antinatalism is an umbrella term for numerous moral dilemmas associated with procreation, each one of them defining it slightly differently; however, all of them devaluate procreation. The most known is argument antinatalism, introduced by D. Benatar in 1997, whose general idea is that “being brought into existence is not a benefit but always a harm”.

The natural drift toward owning offspring has always been perceived as obvious and innate, and for years, it has been strongly socially reinforced [1]. However, recently, due to environmental, economic, social, and health changes, it is not so evident anymore. In the past few years, when antinatalism emerged, it has been more of a philosophical curiosity rather than a significant theory. However, the inevitable deterioration of environmental conditions and recurrent numerous social difficulties, such as the global worsening of people’s mental health, poverty, and the COVID-19 pandemic, induced the discussion of personal responsibility for our planet’s future. Out of this, several types of antinatalism emerged; for example, environmental or local antinatalism [2].

Ecological antinatalism focuses on the irreversible harm that people cause to the environment and, as a consequence, leads to postulates such as: reducing the number of people would slow down the changes, and bringing new lives into the world, which is endangered by environmental catastrophe, is highly immoral. The other one, local antinatalism, states that people who are unable to provide for their children, causing them to mainly experience suffering, should not procreate. Other types of antinatalism include philanthropic and misanthropic antinatalism, the first one stating that living always leads to suffering, whereas the other indicates that people always cause harm to everything that surrounds them, making their lives miserable [3,4].

Inevitably, environmental issues such as climate change, chemical and air pollution, and poor water quality affect human health and wellness. Detrimental substances in the environment increase the risk of diseases, disability, and even death, creating a larger need for better medical care. It becomes particularly important as far as procreation is concerned. At the same time, in the last few years, relatively fewer Polish medical doctors have decided to start their training in pediatrics or obstetrics due to, among other reasons, the work conditions and law environment [5,6,7,8]. Therefore, access to obstetric and pediatric care remains and will remain rather suboptimal, thus elevating risks connected with pre, peri, and postnatal care. These possible pregnancy and birth complications influence subsequent antinatalist views. Moreover, the fear of pregnancy and birth complications are the factors forming antinatalistic views [9]. What is more, ecological issues, industrialization, the development of new technologies, or chronic stress intensify the global deterioration of mental health, which is another emerging problem of today’s medicine [10,11,12].

The majority of reports of antinatalist views remain rather in the field of philosophical discussion [1,13], showing neither scientific data nor scientific conclusions—they usually submit superficial descriptions in the newspapers willingly shared through social media rather than in academic papers. There is a dearth of research describing the participants enrolled in the study, methods of evaluation, or limitations of the study. Finally, most papers fail to analyze any predictors of the phenomenon, as well as foresee its consequences [2], which seem to be the most important in the discussion.

**Figure 1:**
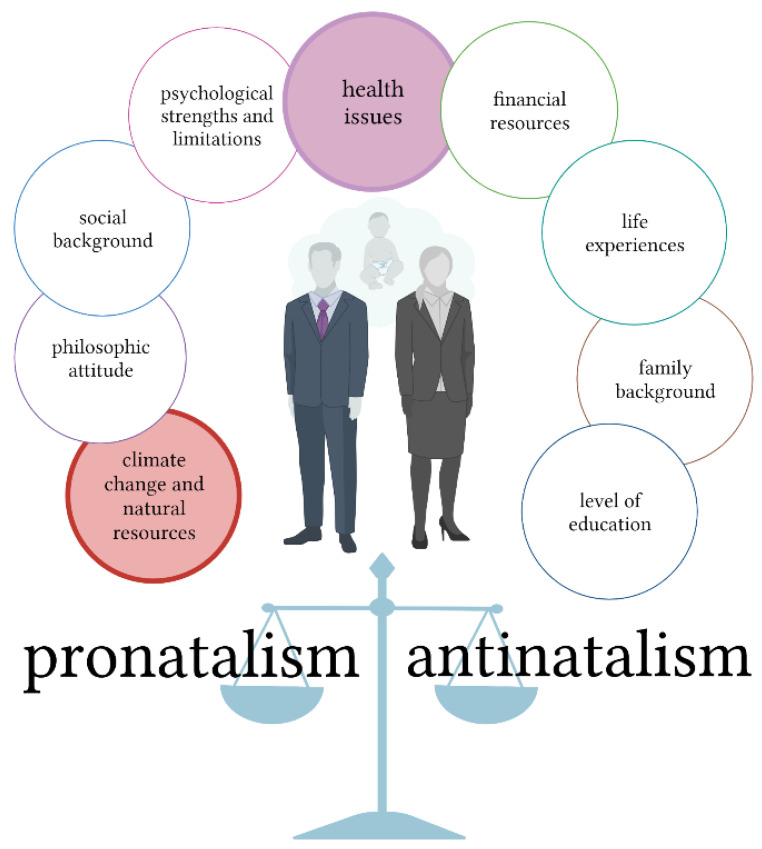
The multifaceted nature of antinatalism. Antinatalism is influenced by a variety of different factors, such as environmental changes, health care issues, philosophic attitude, social background, psychological aspects, financial resources, life experiences, family background, and level of education.

As antinatalism has a multifaced nature and is influenced by a variety of different factors, in this study, we concentrated only on environmental changes and healthcare issues, including mental health. The other aspects of antinatalism, e.g., social, economic, psychological, and philosophical, should be evaluated in other studies to keep the paper more clear and comprehensive for readers (see Figure 1).

Science provides us with a large amount of evidence on how many negative changes the Earth is undergoing, emphasizing the anthropogenic contribution. Within the last thirty years, the globally averaged combined land and ocean surface temperature has gradually increased. The last two decades brought the Greenland and Antarctic ice sheets to lose mass. The sea level has risen, the atmospheric concentrations of carbon dioxide, methane, and nitrous oxide have increased, and the ocean that absorbs the excess of emitted carbon dioxide has acidified [14]. We obtain more and more evidence that climate change will influence human surroundings and wellbeing. The World Health Organization (WHO) projects approximately 250,000 additional deaths per year between 2030 and 2050 as a result of climate change [15]. Besides that, people may be affected by heatwaves, drought, starvation or altered illnesses, and water pollution that leads to various illnesses, e.g., diarrhea or cachexia [16].

Finally, antinatalism is a highly important topic in Poland in which the number of newborns declined from 701, 553 in 1980 to 356, 540 in 2020 [17]. Therefore in the current study, we aimed to characterize antinatalists in the Polish population in terms of the frequency and description of the main reasons behind this phenomenon. Secondly, we decided to investigate the reasons for antinatalism and assess what could change the respondent’s attitude toward having offspring.

## 2. Materials and Methods

### 2.1. Study Design and Participants

The cross-sectional study was performed on the Polish population. The self-administered online questionnaire was available on Google Forms between 19 January 2022 and 25 January 2022. The survey was intentionally published after the Christmas holiday and New Year’s Eve season, which are believed to have a great influence on people’s wellbeing. Participants were invited to take the survey through social media (Facebook, Instagram, and YouTube). All participants were aware of the study conditions and gave informed consent to participate. Confidentiality and anonymity were maintained and no data that could help to identify a responder were collected. The local bioethics committee confirmed that, according to Polish law and good clinical practice regulations, the study does not require the approval of the Bioethics Committee of the Medical University of Lodz (RNN/289/21/KE).

### 2.2. Antinatalism and Pronatalism Definition

In our study, antinatalists were defined as people who do not have children and have no willingness to have them in the future, whereas pronatalists were defined as people who want or/and have children.

### 2.3. Measurement Tools

A four-part questionnaire was prepared using Google Forms (see Supplementary Materials for English and Polish language versions of the questionnaire). The first part concerned general demographic information such as age, gender, income, professional status, the population size of the place of residence, and employment situation. The second part focused on attitudes towards offspring issues. At this stage, the participants, apart from answering questions on their family relations as well as evaluating their attitudes towards parenthood and factors influencing opinions/attitudes/choices on procreation issues—consulted with an experienced clinical psychologist and psychotherapist (A.P.)—were allowed to answer open-ended questions. The third part included the standardized Depression, Anxiety, and Stress Scale-21 Items questionnaire (DASS-21) used to measure the intensity of depression, anxiety, and stress. Each item was rated on a 0–3 scale, so the total scores ranged from 0 to 63. A higher score suggests more severe depression, anxiety, and/or stress symptoms. It was validated for the Polish population [18,19]. The scale has a very good internal consistency (Cronbach’s α: 0.95) that allows for its use in the study. The whole questionnaire is available as Supplement S1.

### 2.4. Data Division

Due to a large amount of obtained data and the multifaceted nature of antinatalism in this paper, we will focus only on health aspects (mental health, obstetric care) and environmental antinatalism (see Figure 1). The social, philosophic, and psychological aspects of antinatalism were intentionally avoided to increase the clarity of the paper.

### 2.5. Statistical Analysis

No data obtained normal distribution (Shapiro–Wilk’s test, *p* > 0.05), and thus, they were reported as a median (1. Quartile–3. Quartile). The independent subgroups’ relationships were assessed using the Mann–Whitney U test. The nominal data, as presented by *n* (%), were analyzed using chi-square test, the chi-square test with Yates’ correction, or Fisher’s test based on the size of the smallest subgroup (*n* ≥ 15, 15 > *n* ≥ 5, 5 > *n*, respectively). Binary logistic regression was performed to assess the facilitators and barriers affecting the willingness to have a child. The regression model (a forward stepwise model) was built based on the univariate analysis and further adjusted to the baseline characteristics of the enrolled subjects (sex, age, income, and university degree). Statistical analysis was performed using STATISTICA 13.1 (TIBCO, Palo Alto, Santa Clara, CA, USA). A level of 5% was used as a significance threshold for all of the results. Moreover, Cronbach’s α was calculated using DATAtab [20].

During the analysis of qualitative data, each respondent’s answer was subjectively analyzed by authors and assigned to certain groups. The most frequently occurring groups, not included in the questionnaire, were additionally mentioned in Section 3.

## 3. Results

### 3.1. Study Group

The study comprised 1240 respondents: including 539 (43.47%) declaring themselves as males and 692 (55.81%) as females, with 706 (56.94%) of them being in a stable relationship. See Table 1 for further details.

Respondents were assigned to four groups according to their willingness to have children and their current offspring/parenthood situation. The first group was composed of people who have at least one child and wanted to have more, the second comprised those who do not have any children but wanted to have at least one, the third group consisted of respondents who have at least one child but do not want to have more, and the last group was represented by antinatalists (see Supplement S2 Table S1 for further details). The most numerous group was group 2. The smallest share of men was observed in group 4. However, the group with the highest mean age turned out to be group 3. See Table 2 for further details.

In further paragraphs, we have chosen the following group assignment: pronatalists (group 1, group 2, and group 3; *n* = 768, 62%) and antinatalists (group 4; *n* = 472, 38%).

### 3.2. Willingness to Have a Child

In the assessed group, 768 (61.94%) revealed their willingness to have a child, in the meaning of both having a child or the desire to have at least one (pronatalists). Antinatalists were represented by 472 (39.06%) respondents. Antinatalist and pronatalist groups did not differ as far as gender, age, and living/staying abroad were concerned. In the group of pronatalists, 356 (46.35%) respondents were male, the median age was 25 (21–29) years, and 720 (95.36%) were living in Poland. In the antinatalists group, 183 (39.77%) respondents were male, the median age was 23 (20–27) years, and 439 (95.64%) were living in Poland. Gender, age, and living/staying abroad did not present statistically significant differences between pronatalist and antinatalist groups.

### 3.3. Factors Discouraging from Having a Child

Further, we attempted to identify factors discouraging from having a child. The most frequent answers were: fear of pregnancy and postpartum complications and fear of a child’s congenital defects/diseases. Among parameters collected in Table 3, the significant difference between groups was determined for fear of pregnancy and postpartum complications. Moreover, other significant differences between the groups were determined by the separation of the female subgroup; see Table 3.

Pronatalists and antinatalists did not differ in DASS-21 subscales; however, female subgroups varied in the level of depression and anxiety (see Table 4).

Additionally, better access to obstetricians, gynecologists, pediatricians, and prenatal diagnostics, as an encouraging factor, did not reveal a significant difference between the groups of pronatalists and antinatalists: 3 (1–4) vs. 3 (2–4.25), respectively, *p* = 0.322. The same phenomenon was observed with a better climate policy and decreasing the risk of the climate crisis: 3 (2–5) vs. 3 (2–5), respectively, *p* = 0.559.

### 3.4. The Influence of Climate Changes on Willingness to Have a Child—Environmental Antinatalism

In the context of climate change, respondents’ answers were significantly different between the groups. Antinatalists considered climate change as a more important cause for deciding not to have a child, comparing with pronatalists. See Table 5 for further details.

### 3.5. Analysis of Facilitating Factors and Barriers to the Desire to Have a Child

We performed a logistic regression model (a forward stepwise model) based on univariate analysis and further adjusted it to the baseline characteristics of the enrolled subjects (sex, age, income, and university degree). The most significant baseline parameters related to salary, education, career, and family were added. The juxtaposition of parameters regarding baseline characteristics and environmental antinatalism allows us to assess more accurately the impact of environmental antinatalism. For example, parameters regarding environmental antinatalism are decreasing the willingness to have a child more than the fear associated with professional duties and their career. See Table 6 for further details.

## 4. Discussion

The research examines factors associated with antinatalistic views and attitudes in the cohort of the Polish population; in particular, factors related to environmental and medical issues. In our study, 39.06% of participants were classified as antinatalists. When compared to pronatalists, they differed significantly in their fear of pregnancy and postpartum complications and attitude towards climate change. Antinatalists considered climate change as a significant reason not to have a child twice as often in comparison with pronatalists. Moreover, the performed binary logistic regression model highlighted the importance of the fear of climate change as an independent factor facilitating an antinatalistic attitude. Regarding females, there were more factors observed that discouraged from having a child, such as: fear of child’s congenital diseases, pregnancy complications, dissatisfaction with medical services, and fear of exacerbation of maternal chronic diseases. Our findings also suggest that anxiety, depression, and stress were not found to be statistically different between pro- and antinatalist groups. However, further analysis revealed that female antinatalists were significantly more depressive and anxious. In our study, antinatalists were represented by approximately 40% of the participants. In the research of Rybińska and Morgan from 2019, 14.7% of women did not decide to have children. However, only 0.3% of them were sure about that throughout their whole lives. The rest of the women, at least for some time in their lives, wanted to have children, and their opinion changed over time [21]. In another study by Neal and Neal conducted in USA in 2022, over one fifth of the population (21.64%) decided to remain childless [22]. Even though our study is partially biased due to the selection of the population from bigger cities with higher education, it correctly illustrates the direction of antinatalism popularization in the society [23].

Our antinatalistic respondents considered climate change as an important factor when deciding to have a child. However, a better climate policy and actions that allow us to decrease the risk of climate collapse were not mentioned as factors that increased their willingness to procreate. What may be the reason for such a phenomenon?

Based on the State of Science Index Survey [24], during the COVID-19 pandemic, Poles’ concern about environmental issues increased in 65% of cases. However, climate change awareness is still evolving in the Polish population; thus, environmental antinatalism, which we focused on, may also not be very widely recognized. According to the Special Eurobarometer on Climate Change [25], only 11% of Polish respondents perceive those changes as the most serious and threatening global matter. The result for the European Union as one was 18%, simultaneously ranking it first as the single most serious problem facing the world as a whole. It was ahead of issues such as poverty, hunger, and lack of drinking water. However, Siña et al. found that, for residents of Lima municipalities, climate change and general environmental issues are perceived as less serious than public safety and water and sanitation services. They proposed that this perception may result from a poor understanding of the concept and its consequences [26].

People experience a variety of emotions towards climate change. Smith and Leiserowitz described their respondents’ reactions as disgusted, worried, hopeful, helpless, angry, and sad when asked about global warming [27]. Only one-third of them admitted to feeling afraid. Stewart developed a new measure of climate change worry, with worry being a core of both anxiety and depression [28]. Searle et al. established associations between climate change distress and depression, anxiety, and stress. However, concerning ecological issues, worry turns out to be a constructive and adaptive response, correlated with positive attitudes and behaviors [29].

Nonetheless, knowledge and negative feelings may not be enough to change peoples’ actions; for instance, Rodríguez-Cruz and Niles studied the population of Puerto Rican’ farmers who, despite awareness of climate change’s impact on their farms and motivation to adapt to changes, did not implement any strategy in their work fields [30]. Di Giusto et al. surveyed students in Taiwan universities and only 28% of their respondents declared to be “very concerned” about climate change, yet merely 11%, in general, reported changing “very much” in their behavior in response to it [31].

Gifford suggested that the human brain is not as rational as previously expected; therefore, climate change seems anabstract, slow, and distant problem. He pointed out several factors that influence people’s ability to act, such as ignorance, optimism bias, or system justification, which make us poorly equipped for environmentalism [32].

Many antinatalist respondents agreed that believing that their potential child will have a “miserable life” should lead to remaining childless, which is in concordance with the study of Schönegger. His research proved that antinatalist views are related to dark triad personality traits (narcissism, Machiavellianism, and psychopathy understood as “tendencies” rather than clinical diagnoses) and a depressive mood. This is despite the primary impression that it may be presented in individuals with high empathy instead due to their concern for the suffering of future generations. The author argues that it might be caused by the divergent character of those views. However, we did not find a difference between antinatalists and pronatalists in the field of depression, anxiety, and stress disorders based only on the DASS-21 scale.

That, however, may not be the case later in life. According to Grundy et al., in Eastern European countries, childlessness and having one child compared with two children indicates a predisposition to having more depressive symptoms. This might be caused by a lack of having close kin for emotional and economic support. Interestingly, this phenomenon was not present in Western Europe [33]. On the other hand, Bonsang et al. suggest that having three or more children versus only two hurts late-life cognition. They argue it by pointing out that, among others, having that many children affects parents’ late-life financial and social resources, thus causing them additional stress [34].

In our study, the highest answers when asked about factors discouraging from having a child were fear of pregnancy and postpartum complications, as well as fear of child’s congenital disorder (including only health and environment-related parameters). This was especially underlined among female respondents.

This is to be expected as the number of reported pregnancy-related deaths in the United States steadily increases from 7.2 deaths per 100,000 live births in 1987 to 17.3 deaths per 100,000 in 2017 [35]. However, the incidence of such a fatal event as maternal death still remains quite low, and the fears associated with pregnancy and childbirth are common around the world, e.g., according to Melender et al., 78 percent of women expressed fears related to pregnancy, childbirth, or both, based on the Finnish population [9].Furthermore, Cetişli et al. determined that, in Turkish society, pregnant women have high levels of anxiety about childbirth and the postpartum period [36].

Another prevalent issue was fear of child’s congenital defects and diseases. Despite the general risk of severe abnormalities remaining relatively low, every pregnant woman must face this possibility [37]. Experiencing the birth of a child with a congenital defect—as shown by Nayeri et al. as an example of a congenital heart defect—is a devastating event that, if left without proper support, may lead to an acute stress disorder and post-traumatic disorder, increasing the risk of sleep and eating disorders [38]. In our study, fear of child’s congenital defect remained rated as discouraging by both antinatalist and pronatalists. Among females declared as being antinatalist, this note was even higher. Along with screening guidelines and studies, such as Nicolaides or Amarin and Akasheh, the risk of congenital defects increases with maternal age [39,40].

The mean age of women bearing their firstborn in Poland has increased from 25.8 in 1990 to 30.5 in 2020 [41]. In our study, the predicted mean age of the first pregnancy is around 29 years old; however, several dozen women could not specify when they would like to have a child, which presumably may increase the predicted mean age of the first pregnancy. It is widely known that the more advanced the age of the mother, the higher the probability of complications—both for her, the fetus, and the newborn. Weight, gestational diabetes, gestational hypertension, a higher probability for induced labor and elective cesarean section, fetal loss, preterm delivery, lower birth weight, longer hospitalization in Neonatal Intensive Care Unit, worse Apagar scores [42,43,44]—all of these must be taken into consideration when taking care of pregnancy in advanced age women. If this trend continues, gynecologic and obstetric care will have to undergo severe changes to meet the needs. Gynecologists, obstetricians, neonatologists, and pediatricians are specialties that are markedly associated with how many infants will be born, affecting the demand for their competencies, and perspectives for work and career development. Nevertheless, our respondents, regardless of if they were declared to be antinatalists or not, did not differ in their opinion on better access to healthcare—in both groups, this issue was graded as irrelevant with female respondents rating it, respectively, higher.

From an obstetric point of view, it is also very important to underline that climate change will directly influence not only pregnancy outcomes but also fertility itself. An elevated ambient temperature may lead to more pre-term births, a low birth weight, stillbirth, and gestational diabetes in mothers [45]. Heat shock may affect reproductive tissues mostly by oxidative stress and unregulated production of free radicals, reactive oxygen species, and reactive nitrogen species. This can lead to the impairment of gametogenesis and mitochondrial malfunctions, as well as an increased germ cell apoptosis [17]. Moreover, air pollution may lead to the development of various congenital defects [46]. That is another challenge that gynecologic care will have to face.

Simultaneously, it is also proven that voluntary family planning can influence and benefit climate change, mostly through slowing future population growth, especially in low- and middle-income countries [47]; thus, the connection between birth rate and climate change is indisputable.

## 5. Advantages and Limitations

This is one of the very first studies exploring concerns about having or not having children among the Polish population. It has many references to current and widely discussed concerns that will impact the future of not only the single nation but eventually the whole world. It uses the widely recognized score to measure depression, anxiety, and stress states, which allows it to be easily reproduced. Moreover, our study gave a special insight into the personal beliefs and thoughts of our respondents, which may turn out to be useful when rethinking family planning politics or social support policy.

We identified the following limitations of the study: firstly, results were based on the online survey that was available only for people using the Internet and social media, which could be responsible for selection bias. Further, the topic of the study could encourage antinatalists to fully fill out the questionnaire more than pronatalists, even though we avoided using the word “antinatalism” in the title of the survey, which was named “Influence of climate change, depression and anxiety disorders on the desire to have children in Poland”. Finally, we did not assess the social approval parameter.

## 6. Conclusions

Antinatalism, as a dilemma in the sense of existence and procreation, is indisputably receiving more and more attention. In our study, around 38% of Polish participants wanted to remain childless. Antinatalism views included environmental antinatalism, depression, anxiety, and medical factors, including fear of a child’s congenital diseases and pregnancy complications. However, the analysis did not include economic, social, and philosophical issues. Paradoxically, better access to medical services and changes in climate politics were not seen as significant enough to encourage society to decide to have offspring. The explanation of that phenomenon needs further studies.

## Figures and Tables

**Table 1:** Study group characterization (*n* = 1240).

	Participants
Total; *n*	1240
Male; *n* (%)	539 (43.47%)
Median age	24 (21–28)
Lives in Poland	1183 (95.40%)
Stayed abroad for more than 6 months	155 (12.50%)
Population of the place of employment/study; *n* (% of complete data):	
City > 500,000 residents	631 (50.86%)
City > 250,000 residents	145 (11.69%)
City > 100,000 residents	159 (12.82%)
City > 50,000 residents	75 (6.05%)
City < 50,000 residents	148 (11.95%)
Countryside	82 (6.63%)
Place of residence as a child: *n* (% of complete data)	
City > 500,000 residents	257 (20.73%)
City > 250,000 residents	90 (7.26%)
City > 100,000 residents	139 (11.21%)
City > 50,000 residents	136 (10.97%)
City < 50,000 residents	297 (23.95%)
Countryside	321 (25.88%)
Formal education:	
Higher	581 (46.85%)
Complete secondary education	496 (40%)
Incomplete secondary education	105 (8.47%)
Professional training	20 (1.62%)
Primary education	38 (3.06%)
Income:	
<0.3 national average	82 (6.61%)
0.3–0.5 national average	234 (18.87%)
0.5–1.0 national average	536 (43.23%)
1.0–1.5 national average	235 (18.95%)
>1.5 national average	153 (12.34%)
Stable relationship (at least 6 months)	706 (56.94%)

**Table 2:** The basic characteristic of groups.

	1	2	3	4
Total	55 (4.45%)	637 (51.38%)	76 (6.14%)	472 (38.07%)
Male	31 (56.36%)	280 (44.96%)	45 (59.21%)	183 (39.77%)
Median age	30 (28–35.5)	24 (21–27)	37.5 (33–42.5)	23 (20–27)
(a) how many children do they have?	1 (1–1)	N.A.	2 (1–2)	N.A.
(b) when approximately do they want to have children (in years)? Among females	1 (0–2) 2 (0–3)	5 (2–7) 5 (2–7)	N.A. N.A.	N.A. N.A.
(c) what percentage of them consider adoption [% of responses yes/no]	11 (27.50%)	188 (51.37%)	4 (5.80%)	113 (34.14%)
(d) percentage of infertility [% of responses yes/no]	8 (17.78%)	20 (17.86%)	4 (5.88%)	7 (6.80%)

Legend: N.A., non applicable. Nominal data are presented as *n* (%), whereas other data are present as median with interquartile range.

**Table 3:** Parameters discouraging from having a child. 1—does not discourage at all, 2—rather does not discourage, 3—has no opinion, 4—rather discourages, 5—definitely discourages. Data are presented as median (1. Quartile–3. Quartile).

	The Willingness to Have a Child		*p*-Value
	Pronatalists	Antinatalists	
Fear of child’s congenital defects/diseases.	4 (3–5)	4 (3–5)	0.664
Among females:	4 (3–5)	5 (4–5)	**<0.001**
Dissatisfaction with the medical services (e.g., access to gynecologists, obstetricians, pediatricians, and prenatal diagnosis)	4 (2–5)	4 (2–4.5)	0.488
Among females:	4 (2–5)	4 (3–5)	**<0.001**
Fear that climate changes will force the offspring to live on the destroyed planet (e.g., increased global average temperature, rising seas levels, extreme weather, resources conflicts).	3 (2–5)	3 (2–5)	0.843
Fear of pregnancy and postpartum complications.	4 (3–5)	4 (2–5)	**0.035**
Among females:	4 (3–5)	5 (4–5)	**<0.001**
Fear of exacerbation of maternal chronic diseases.	3 (2–4.5)	3 (2–4)	0.785
Among females:	3 (2–4)	4 (3–5)	**<0.001**

Bold—statistically significant results.

**Table 4:** DASS-21 in the context of the willingness to have a child.

	The Willingness to Have a Child		*p*-Value
	Pronatalists	Antinatalists	
Depression Among females:	9 (4–14) 8 (4–14)	8 (4–14) 12 (6–17)	0.357 **<0.001**
Anxiety Among females:	5 (2–10) 6 (3–11)	6 (2–10) 8 (4–12)	0.570 **0.013**
Stress Among females:	9 (5–13) 10 (6–14)	9 (5–13) 11 (7.5–15)	0.900 0.064

Bold—statistically significant results.

**Table 5:** Parameters affecting willingness to have a child in the context of environmental changes.

	The Willingness to Have a Child		*p*-Value
	Pronatalists	Antinatalists	
Do you think that environmental causes (increasing average temperature on Earth, extreme weather, rising seas levels, climate migration) are important reasons for not deciding to have children?	276 (35.49%)	289 (61.23%)	**<0.001**
Do you think that environmental causes present an exclusively sufficient reason to decide to not have a child?	146 (19.01%)	211 (44.70%)	**<0.001**
Humans cause so much harm—to other humans, animals, and the environment—that it is wrong to procreate.	2 (1–3)	3 (2–4)	0.743
I fear the climate disaster and the environmental conditions that my kids will have to/may live in.	4 (2–4)	4 (3–5)	0.726
People deciding to bear children while facing climate change are irresponsible.	1 (1–2)	3 (2–4)	0.307
The Earth is overpopulated, and restricted resources do not allow us to reproduce uncontrollably.	3 (1–4)	4 (3–5)	0.549
I am afraid that, if I decided to have children, they would witness terrible consequences of climate change.	3 (2–4)	4 (3–5)	0.964

Bold—statistically significant results; nominal data are present as *n* (%), whereas other data are present as median with interquartile range.

**Table 6:** The logistic regression model evaluated the impact of tested parameters on the willingness to have a child and was adjusted to the baseline characteristics of the enrolled subjects.

The Binary Logistic Regression Model			
	OR	95%CI	*p*-Value
Intercept	4.280	1.912–9.581	**<0.001**
The willingness to develop professional career	0.372	0.316–0.438	**<0.001**
The willingness to meet the right partner	1.735	1.508–1.997	**<0.001**
The fear of unfavorable climate change	0.512	0.366–0.715	**<0.001**
Higher income	1.347	1.194–1.519	**<0.001**
Higher education	1.673	1.214–2.307	**<0.001**
The fear associated with professional duties and career	0.827	0.727–0.941	**<0.001**
The belief that environmental causes are independent reason to decide not to have a child	0.647	0.451–0.927	**<0.001**

Legend: bold—statistically significant results, CI—confidence interval, OR—odds ratio.

## Data Availability

Data available on request due to restrictions eg privacy or ethical.

## Supplementary Materials

The following supporting information can be downloaded at: https://www.mdpi.com/article/10.3390/ijerph192013575/s1, Supplement S1, Survey: Influence of climate change, depression and anxiety disorders on the desire to have children in Poland; Supplement S2, Table S1: Criteria for group assignment.
