# Supplementary material for: The Causes and Role of Antinatalism in Poland in the Context of Climate Change, Obstetric Care, and Mental Health
Source: Int J Environ Res Public Health. 2022 Oct 20;19(20):13575. doi: 10.3390/ijerph192013575 (PMC9602747; doi:10.3390/ijerph192013575)
Supplement: Supplementary file 1 [file ijerph-19-13575-s001.zip › Supplement S1, Survey.pdf]

Wpływ zmian klimatycznych, depresji i zaburzeń lękowych na chęć posiadania potomstwa w Polsce

Zapraszamy do wypełnienia ankiety mającej na celu zbadanie, które czynniki wpływają na podejmowanie decyzji o posiadaniu bądź nieposiadaniu potomstwa w kontekście zmian klimatycznych, lęku, stresu i depresji.

Jest to dobrowolne, anonimowe badanie ankietowe przeprowadzone przez zespół naukowców z Uniwersytetu Medycznego w Łodzi. Ankieta składa się z trzech części — pierwszej ogólnej, drugiej dotyczącej chęci posiadania potomstwa oraz trzeciej oceniającej nasilenie stresu, lęku i niepokoju (standardowy kwestionariusz DASS-21). Warunkiem przystąpienia do ankiety jest ukończenie 18. roku życia. Wypełnienie ankiety jest równoznaczne z wyrażeniem zgody na wzięcie udziału w badaniu oraz przetwarzanie danych zebranych w toku badania. Zgodę na badanie można wycofać w każdej chwili jego trwania. Wypełnienie ankiety powinno Ci zająć około 10 minut. Badanie zostało zaopiniowanie przez Komisję Bioetyczną przy Uniwersytecie Medycznym w Łodzi, numer decyzji RNN/289/21/KE.

Postaraj się wypełnić ankietę jak najrzetelniej i szczerze.

*Wymagane

Pytania ogólne/ Postawowe informacje

W tej sekcji chcielibyśmy jak najwięcej się o Tobie dowiedzieć — znajdziesz tutaj podstawowe pytania o płeć, wiek, miejsce zamieszkania i strukturę rodziny, które pozwolą nam scharakteryzować grupę respondentów.

1. Płeć *

Zaznacz tylko jedną odpowiedź.

- ◯ Mężczyzna
- ◯ Kobieta
- ◯ Inne: ____________________

2. Wiek (wpisz liczbę) *

____

3. Czy mieszkasz w Polsce? *

Zaznacz tylko jedną odpowiedź.

- Tak, mieszkam w Polsce.
- Nie, mieszkam za granicą.

4. Czy kiedykolwiek mieszkałeś/mieszkałaś poza Polską dłużej niż 6 miesięcy? *

Zaznacz tylko jedną odpowiedź.

- Tak
- Nie

5. W jakiej miejscowości pracujesz/studiujesz/najczęściej przebywasz? *

Zaznacz tylko jedną odpowiedź.

- Miasto powyżej 500 tys osób
- Miasto powyżej 250 tys osób
- Miasto powyżej 100 tys osób
- Miasto powyżej 50 tys osób
- Miasto poniżej 50 tys osób
- Wieś

6. W jakiej miejscowości się wychowałeś/wychowałaś? *

Zaznacz tylko jedną odpowiedź.

- Miasto powyżej 500 tys osób
- Miasto powyżej 250 tys osób
- Miasto powyżej 100 tys osób
- Miasto powyżej 50 tys osób
- Miasto poniżej 50 tys osób
- Wieś

7. Jakie masz wykształcenie? *

Zaznacz tylko jedną odpowiedź.

- Podstawowe
- Zawodowe
- Średnie niepełne (brak matury)
- Średnie pełne
- Wyższe

8. Czy nadal studiujesz? *

Zaznacz tylko jedną odpowiedź.

- Tak
- Nie

9. Czy masz stałą pracę? *

Zaznacz tylko jedną odpowiedź.

- Tak
- Nie
- Nie, jestem na rencie/zwolnieniu/emeryturze
- Nadal studiuję/uczę się

10. Jaki jest przeciętny miesięczny dochód netto ("na rękę") przypadający na jednego członka Twojej rodziny? *

Zaznacz tylko jedną odpowiedź.

- <1 250 zł
- 1 250 - 2 250 zł
- 2 250 - 4 250 zł
- 4 250 - 6 250 zł
- > 6 250 zł

11. Czy jesteś obecnie w stałym związku? (trwa dłużej niż 6 miesięcy, albo został sformalizowany) *

Zaznacz tylko jedną odpowiedź.

- Tak
- Nie

12. Jeśli jesteś w stałym związku, od ilu lat on trwa? (jeśli nie jesteś wpisz "nie dotyczy"; jeśli trwa krócej niż rok wpisz "<1") *

____

13. Jeśli jesteś w stałym związku, czy jest on sformalizowany? *

Zaznacz tylko jedną odpowiedź.

- Tak
- Nie
- Nie jestem w stałym związku

14. Jeśli jesteś w nieformalnym związku, czy planujesz jego sformalizowanie? *

Zaznacz tylko jedną odpowiedź.

- Tak
- Nie
- Wolę nie odpowiadać na to pytanie
- Nie jestem w stałym związku
- Nie dotyczy, jestem w sformalizowanym związku

15. Czy należysz do jakiegoś wyznania/religii? *

Zaznacz tylko jedną odpowiedź.

- Tak
- Formalnie należę, ale nie utożsamiam się
- Nie
- Wolę nie odpowiadać na to pytanie

16. Czy masz rodzeństwo? *

Zaznacz tylko jedną odpowiedź.

- Tak, biologiczne
- Tak, przyrodnie (jeden wspólny rodzic)
- Tak, adopcyjne
- Tak, mieszane
- Nie

17. Czy pozostajesz w stałym kontakcie ze swoją najbliższą rodziną (rodzice, rodzeństwo, dziadkowie) lub jej częścią? *

Zaznacz tylko jedną odpowiedź.

- Tak
- Tak, ale mamy ze sobą kontakt tylko sporadycznie
- Nie utrzymuję kontaktu

18. Swoje relacje z rodzicami w dzieciństwie oceniam jako: *

Zaznacz tylko jedną odpowiedź.

- Bardzo dobre
- Dobre
- Średnie
- Złe
- Bardzo złe
- Nie miałem/miałam z nimi relacji

19. Swoje relacje z rodzeństwem w dzieciństwie oceniam jako: *

Zaznacz tylko jedną odpowiedź.

- Bardzo dobre
- Dobre
- Średnie
- Złe
- Bardzo złe
- Nie miałem/miałam z nimi relacji

20. Swoje relacje z rodzicami w życiu dorosłym oceniam jako: *

Zaznacz tylko jedną odpowiedź.

- Bardzo dobre
- Dobre
- Średnie
- Złe
- Bardzo złe
- Nie mam z nimi relacji

21. Swoje relacje z rodzeństwem w życiu dorosłym oceniam jako: *

Zaznacz tylko jedną odpowiedź.

- Bardzo dobre
- Dobre
- Średnie
- Złe
- Bardzo złe
- Nie mam z nimi relacji

22. Oceniam siebie jako: *

Zaznacz tylko jedną odpowiedź.

- optymistę/optymistkę
- raczej optymistę/optymistkę
- realistę/realistkę
- raczej pesymistę/pesymistkę
- pesymistę/pesymistkę

23. Czy kiedykolwiek zdiagnozowano u Ciebie zaburzenia depresyjne, albo zaburzenia lękowe? *

Zaznacz tylko jedną odpowiedź.

- Tak, zaburzenia depresyjne (w tym afektywne)
- Tak, zaburzenia lękowe
- Tak, zarówno zaburzenia depresyjne jak i lękowe
- Nie

Ocena postaw wobec spraw dzietności

Ta część skupia się na określeniu Twojego stosunku do posiadania dzieci oraz opisaniu ewentualnych obaw i przekonań z tym związanych.

24. Ile masz dzieci? (podaj liczbę, jeśli nie masz dzieci wpisz 0) *

____

25. Czy są to Twoje biologiczne dzieci? *

Zaznacz tylko jedną odpowiedź.

- Tak, są to moje biologiczne dzieci
- Nie, są adoptowane/przysposobione/przebywają w pieczy zastępczej
- Część jest biologiczna, pozostałe adoptowane/przysposobione/przebywające w pieczy zastępczej
- Nie mam dzieci

26. Czy planujesz kiedykolwiek mieć biologiczne dzieci/zdecydować się na kolejne dziecko? *

Zaznacz tylko jedną odpowiedź.

- Tak, na pewno chcę
- Tak, raczej chcę
- Nie, raczej nie
- Nie, na pewno nie

27. Za ile lat planujesz mieć swoje biologiczne dziecko? (jeśli nie planujesz/nie wiesz wpisz "nie dotyczy"; jeśli za mniej niż rok wpisz "<1") *

____

28. Czy rozważasz adopcję? *

Zaznacz tylko jedną odpowiedź.

- Tak
- Nie
- Nie wiem

29. Czy w Twoim związku występuje problem bezpłodności? (Bezpłodność to niemożność zajścia w ciążę pomimo współżycia z częstością 3-4 stosunków tygodniowo bez stosowania jakiejkolwiek antykoncepcji przez 12 miesięcy) *

Zaznacz tylko jedną odpowiedź.

- Tak
- Nie
- Nie wiem
- Nie dotyczy

W tej części ankiety wymienione zostaną czynniki potencjalnie zniechęcające do posiadania potomstwa. Proszę oceń w jakim stopniu poniższe czynniki dotyczą Ciebie.

1 - wcale nie zniechęca
2 - raczej nie zniechęca
3 - nie mam zdania
4 - raczej zniechęca
5 - zdecydowanie zniechęca

30. Niesatysfakcjonująca sytuacja ekonomiczna. *

Zaznacz tylko jedną odpowiedź.

	1	2	3	4	5	
wcale nie zniechęca						zdecydowanie zniechęca

31. Obawa przed wrodzonymi wadami/chorobami dziecka. *

Zaznacz tylko jedną odpowiedź.

	1	2	3	4	5	
wcale nie zniechęca						zdecydowanie zniechęca

32. Niezadowolenie z opieki zdrowotnej (np. dostęp do ginekologów, położników, pediatrów i diagnostyki prenatalnej). *

Zaznacz tylko jedną odpowiedź.

	1	2	3	4	5	
wcale nie zniechęca	◯	◯	◯	◯	◯	zdecydowanie zniechęca

33. Obawa przed zmianami środowiskowymi, które sprawią, że potomstwo będzie musiało żyć na zniszczonej planecie (np. podniesienie średniej temperatury na Ziemi, podniesienie poziomu wód, skrajne zjawiska pogodowe, walka o zasoby). *

Zaznacz tylko jedną odpowiedź.

	1	2	3	4	5	
wcale nie zniechęca	◯	◯	◯	◯	◯	zdecydowanie zniechęca

34. Brak partnerów, z którymi warto posiadać potomstwo. *

Zaznacz tylko jedną odpowiedź.

	1	2	3	4	5	
wcale nie zniechęca	◯	◯	◯	◯	◯	zdecydowanie zniechęca

35. Obawa przed powikłaniami związanymi z ciążą i połogiem. *

Zaznacz tylko jedną odpowiedź.

	1	2	3	4	5	
wcale nie zniechęca	◯	◯	◯	◯	◯	zdecydowanie zniechęca

36. Obawa przed zaostrzeniem chorób przewlekłych u matki. *

Zaznacz tylko jedną odpowiedź.

	1	2	3	4	5	
wcale nie zniechęca	◯	◯	◯	◯	◯	zdecydowanie zniechęca

37. Obowiązki związane z posiadaniem potomstwa. *

Zaznacz tylko jedną odpowiedź.

	1	2	3	4	5	
wcale nie zniechęca	◯	◯	◯	◯	◯	zdecydowanie zniechęca

38. Obawy związane z obowiązkami zawodowymi i karierą. *

Zaznacz tylko jedną odpowiedź.

	1	2	3	4	5	
wcale nie zniechęca	◯	◯	◯	◯	◯	zdecydowanie zniechęca

39. Obawa przed niebyciem gotowym/gotową na posiadanie potomstwa. *

Zaznacz tylko jedną odpowiedź.

	1	2	3	4	5	
wcale nie zniechęca	◯	◯	◯	◯	◯	zdecydowanie zniechęca

40. Inne czynniki, które zniechęcają mnie do posiadania potomstwa to: (jeśli nie dotyczy wpisz "brak"). *

W tej części ankiety wymienione zostaną potencjalne czynniki mogące przekonać do posiadania potomstwa. Proszę oceń w jakim stopniu poniższe czynniki dotyczą Ciebie.

1 - wcale nie przekonuje
2 - raczej nie przekonuje
3 - nie mam zdania
4 - raczej przekonuje
5 - zdecydowanie przekonuje

41. Zwiększenie socjalnych świadczeń na dziecko. *

Zaznacz tylko jedną odpowiedź.

	1	2	3	4	5	
wcale nie przekonuje	⬭	⬭	⬭	⬭	⬭	zdecydowanie przekonuje

42. Zmniejszenie podatków dla osób wychowujących dzieci. *

Zaznacz tylko jedną odpowiedź.

	1	2	3	4	5	
wcale nie przekonuje	⬭	⬭	⬭	⬭	⬭	zdecydowanie przekonuje

43. Wzrost zarobków. *

Zaznacz tylko jedną odpowiedź.

	1	2	3	4	5	
wcale nie przekonuje						zdecydowanie przekonuje

44. Poprawa polityki środowiskowej i obniżanie ryzyka katastrofy klimatycznej. *

Zaznacz tylko jedną odpowiedź.

	1	2	3	4	5	
wcale nie przekonuje						zdecydowanie przekonuje

45. Lepszy dostęp do opieki zdrowotnej (specjaliści położnictwa, ginekologii, pediatrii; diagnostyka prenatalna). *

Zaznacz tylko jedną odpowiedź.

	1	2	3	4	5	
wcale nie przekonuje						zdecydowanie przekonuje

46. Zmiany prawne dające więcej przywilejów osobom decydującym się na posiadanie potomstwa. *

Zaznacz tylko jedną odpowiedź.

	1	2	3	4	5	
wcale nie przekonuje						zdecydowanie przekonuje

47. Liberalizacja prawa aborcyjnego. *

Zaznacz tylko jedną odpowiedź.

	1	2	3	4	5	
wcale nie przekonuje	◯	◯	◯	◯	◯	zdecydowanie przekonuje

48. Poznanie właściwego partnera. *

Zaznacz tylko jedną odpowiedź.

	1	2	3	4	5	
wcale nie przekonuje	◯	◯	◯	◯	◯	zdecydowanie przekonuje

49. Dłuższy/lepiej płatny urlop rodzicielski. *

Zaznacz tylko jedną odpowiedź.

	1	2	3	4	5	
wcale nie przekonuje	◯	◯	◯	◯	◯	zdecydowanie przekonuje

50. Inne czynniki które mogłyby mnie przekonać do zdecydowania się na posiadanie potomstwa to: (jeśli nie dotyczy wpisz "brak") *

51. Czy Tobie albo komuś w Twojej rodzinie urodziło się dziecko z wadami wrodzonymi? *

Zaznacz tylko jedną odpowiedź.

- Tak
- Nie

52. Wybierze stwierdzenia które najtrafniej oddają Twój stosunek do przewidywanych zmian klimatycznych/środowiskowych takich jak wzrastająca średnia temperatura na Ziemi i podnoszący się poziom wód? *

Zaznacz wszystkie właściwe odpowiedzi.

- [ ] Bardzo mnie to martwi, obawiam się katastrofy klimatycznej
- [ ] Raczej się tym przejmuję
- [ ] Trochę się tym przejmuję
- [ ] Wcale się tym nie przejmuję
- [ ] Staram się o tym nie myśleć
- [ ] Przewidywania te oparte są na błędnych założeniach

53. Czy uważasz przyczyny środowiskowe (wzrastająca średnia temperatura na Ziemi, gwałtowne zjawiska pogodowe, podnoszący się poziom wód, migracja ludności na tle zmieniających się warunków życia) za istotną przyczynę niedecydowania się na potomstwo? *

Zaznacz tylko jedną odpowiedź.

- Tak
- Nie

54. Czy uważasz, że wyłącznie przyczyny środowiskowe są wystarczającym powodem, aby nie decydować się na posiadanie potomstwa? *

Zaznacz tylko jedną odpowiedź.

- Tak
- Nie

W następnych pytaniach spróbuj określić w jakim stopniu zgadzasz się z przedstawionymi tezami.

1 - zdecydowanie się nie zgadzam
2 - raczej się nie zgadzam
3 - nie mam zdania
4 - raczej się zgadzam
5 - zdecydowanie się zgadzam

55. Ludzie wyrządzają wiele zła — innym ludziom, zwierzętom oraz środowisku — tak wiele, że rozmnażanie się jest czymś złym. *

Zaznacz tylko jedną odpowiedź.

	1	2	3	4	5	
zdecydowanie się nie zgadzam	◯	◯	◯	◯	◯	zdecydowanie się zgadzam

56. Ciąża to niesamowity i bardzo ubogacający czas w życiu kobiety, każda kobieta powinna chociaż raz w życiu jej doświadczyć. *

Zaznacz tylko jedną odpowiedź.

	1	2	3	4	5	
zdecydowanie się nie zgadzam	◯	◯	◯	◯	◯	zdecydowanie się zgadzam

57. Myślący o przyszłości rodzice, którzy uważają, że ich dziecko będzie miało smutne życie, nie powinni się rozmnażać. *

Zaznacz tylko jedną odpowiedź.

	1	2	3	4	5	
zdecydowanie się nie zgadzam	◯	◯	◯	◯	◯	zdecydowanie się zgadzam

58. Posiadanie dziecka jest równoznaczne z sukcesem życiowym. *

Zaznacz tylko jedną odpowiedź.

	1	2	3	4	5	
zdecydowanie się nie zgadzam	⬭	⬭	⬭	⬭	⬭	zdecydowanie się zgadzam

59. Lepiej nie istnieć niż istnieć, ponieważ tylko istniejąc, możemy doświadczać bólu i cierpienia — jeśli ktoś nie istnieje, nie może cierpieć. *

Zaznacz tylko jedną odpowiedź.

	1	2	3	4	5	
zdecydowanie się nie zgadzam	⬭	⬭	⬭	⬭	⬭	zdecydowanie się zgadzam

60. Dziecko jest najlepszym dowodem szczęśliwego i pełnego miłości związku. *

Zaznacz tylko jedną odpowiedź.

	1	2	3	4	5	
zdecydowanie się nie zgadzam	⬭	⬭	⬭	⬭	⬭	zdecydowanie się zgadzam

61. Obawiam się katastrofy klimatycznej i tego, w jakich warunkach przyjdzie/przyszłoby żyć moim dzieciom. *

Zaznacz tylko jedną odpowiedź.

	1	2	3	4	5	
zdecydowanie się nie zgadzam	⬭	⬭	⬭	⬭	⬭	zdecydowanie się zgadzam

62. Ludzie decydujący się na dzieci w obliczu zmian klimatycznych są nieodpowiedzialni. *

Zaznacz tylko jedną odpowiedź.

	1	2	3	4	5	
zdecydowanie się nie zgadzam	⬭	⬭	⬭	⬭	⬭	zdecydowanie się zgadzam

63. Wolę skupić się na rozwoju, samorealizacji i swojej karierze niż powiększaniu rodziny. *

Zaznacz tylko jedną odpowiedź.

	1	2	3	4	5	
zdecydowanie się nie zgadzam	⬭	⬭	⬭	⬭	⬭	zdecydowanie się zgadzam

64. Ziemia jest przeludniona, ograniczone zasoby nie pozwalają nam na niekontrolowane rozmnażanie. *

Zaznacz tylko jedną odpowiedź.

	1	2	3	4	5	
zdecydowanie się nie zgadzam	⬭	⬭	⬭	⬭	⬭	zdecydowanie się zgadzam

65. Niedecydowanie się na potomstwo to po prostu wymówka w celu ucieczki przed braniem na siebie odpowiedzialności. *

Zaznacz tylko jedną odpowiedź.

	1	2	3	4	5	
zdecydowanie się nie zgadzam	⬭	⬭	⬭	⬭	⬭	zdecydowanie się zgadzam

66. Obawiam się, że jeśli zdecydowałbym/zdecydowałabym się na potomstwo, to doświadczyłoby ono strasznych skutków zmian klimatycznych. *

Zaznacz tylko jedną odpowiedź.

	1	2	3	4	5	
zdecydowanie się nie zgadzam	⬭	⬭	⬭	⬭	⬭	zdecydowanie się zgadzam

67. Zawsze chciałem/chciałam zostać rodzicem. *

Zaznacz tylko jedną odpowiedź.

	1	2	3	4	5	
zdecydowanie się nie zgadzam	⬭	⬭	⬭	⬭	⬭	zdecydowanie się zgadzam

Ocena nasilenia stresu, lęku i niepokoju

W tej części przyjrzymy się nasileniu myśli i objawów związanych z zaburzeniami nastroju.

Poniższe pytania pochodzą z kwestionariusza DASS-21 - standaryzowanej Skali Depresji, Lęku oraz Stresu. Proszę przeczytać każde zdanie i zaznaczyć odpowiedź, która sugeruje u Ciebie nasilenie danego w przeciągu ostatniego tygodnia.
Prosimy postarać się być jak najbardziej szczerym i zgodnym ze swoimi odczuciami.

Skala ocen jest następująca i dotyczy ostatniego tygodnia:
0 – Nie dotyczy mnie w ogóle – NIGDY
1 – Dotyczy mnie w pewnym stopniu lub raz na jakiś czas – CZASAMI
2 – Dotyczy mnie w znacznym stopniu lub przez dłuższą część czasu – CZĘSTO
3 – Dotyczy mnie w bardzo dużym stopniu lub przez większość czasu – ZAWSZE/PRAWIE ZAWSZE

68. 1. Trudno było mi się odprężyć. *

Zaznacz tylko jedną odpowiedź.

0	1	2	3
⬭	⬭	⬭	⬭

69. 2. Odczuwałam/-em suchość w ustach. *

Zaznacz tylko jedną odpowiedź.

0	1	2	3
⬭	⬭	⬭	⬭

70. 3. Nie mogłam/-em doświadczyć pozytywnych uczuć. *

Zaznacz tylko jedną odpowiedź.

0	1	2	3
⬭	⬭	⬭	⬭

71. 4. Odczuwałam/-em trudność w oddychaniu (np. duszności, przyspieszony oddech bez wcześniejszego wysiłku fizycznego). *

Zaznacz tylko jedną odpowiedź.

0	1	2	3
⬭	⬭	⬭	⬭

72. 5. Trudno było mi znaleźć inicjatywę do działania. *

Zaznacz tylko jedną odpowiedź.

0	1	2	3
⬭	⬭	⬭	⬭

73. 6. Miałam/-em skłonności do przesadnego reagowania na różne sytuacje. *

Zaznacz tylko jedną odpowiedź.

0	1	2	3

74. 7. Odczuwałam/-em drżenie np. rąk. *

Zaznacz tylko jedną odpowiedź.

0	1	2	3

75. 8. Czułam/-em, że zużywam dużo nerwowej energii. *

Zaznacz tylko jedną odpowiedź.

0	1	2	3

76. 9. Byłam/-em przestraszony sytuacjami, gdzie mogłem spanikować lub ośmieszyć się. *

Zaznacz tylko jedną odpowiedź.

0	1	2	3

77. 10. Czułam/-em, że nie ma przede mną przyszłości, nic mnie nie czeka w życiu. *

Zaznacz tylko jedną odpowiedź.

0	1	2	3

78. 11. Czułam/-em się pobudzona/-y (niespokojna/-y). *

Zaznacz tylko jedną odpowiedź.

0	1	2	3

79. 12. Trudno było mi się zrelaksować. *

Zaznacz tylko jedną odpowiedź.

0	1	2	3

80. 13. Czułam/-em się smutna/-y i przygnębiona/-y. *

Zaznacz tylko jedną odpowiedź.

0	1	2	3

81. 14. Byłam/-em nietolerancyjna/-y do wszystkiego, co uniemożliwiało mi wzięcie udziału w tym, co robiłem/am. *

Zaznacz tylko jedną odpowiedź.

0	1	2	3
⬭	⬭	⬭	⬭

82. 15. Byłam/-em bliski wpadnięciu w panikę. *

Zaznacz tylko jedną odpowiedź.

0	1	2	3
⬭	⬭	⬭	⬭

83. 16. Nie byłam/-em zdolny do odczuwania entuzjazmu. *

Zaznacz tylko jedną odpowiedź.

0	1	2	3
⬭	⬭	⬭	⬭

84. 17. Czułam/-em, że jestem niewiele warta/-y. *

Zaznacz tylko jedną odpowiedź.

0	1	2	3
⬭	⬭	⬭	⬭

85. 18. Czułam/-em, że jestem raczej przewrażliwiona/-y. *

Zaznacz tylko jedną odpowiedź.

0	1	2	3
⬭	⬭	⬭	⬭

86. 19. Zdałam/-em sobie sprawę z pracy mojego serca mimo braku wysiłku fizycznego (np. przyspieszone bicie serca, zanik bicia serca). *

Zaznacz tylko jedną odpowiedź.

0	1	2	3
⬭	⬭	⬭	⬭

87. 20. Czułam/-em się przestraszona/-y bez powodu. *

Zaznacz tylko jedną odpowiedź.

0	1	2	3
⬭	⬭	⬭	⬭

88. 21. Czułam/-em, że życie nie ma sensu. *

Zaznacz tylko jedną odpowiedź.

0	1	2	3
⬭	⬭	⬭	⬭

Follow-up

TA CZĘŚĆ BADANIA NIE JEST WYMAGANA DO JEGO UKOŃCZENIA. MOŻNA JĄ POMINĄĆ.

W dalszej części badania chcielibyśmy przyjrzeć się temu, jak zmieniają się w czasie Wasze poglądy na chęć posiadania potomstwa w Polsce. Aby to zrobić, prosimy Was o wygenerowanie kodu, który pozwoli nam połączyć wasze kolejne odpowiedzi z dziś wypełnioną ankietą.

Kod składać się będzie z:
pierwsza litera imienia
druga litera nazwiska
dzień urodzenia
ostatnia cyfra peselu
miesiąc urodzenia (styczeń - 01, luty - 02, ... , grudzień - 12)
dwie ostatnie litery nazwiska

Dla przykładu:
JOANNA KIELECKA; 98012345668
=> JI23801KA

W przypadku jakichkolwiek pytań i wątpliwości prosimy o kontakt na maila: ankieta.antynatalizm@gmail.com

89. Twój kod

____

Ta treść nie została utworzona ani zatwierdzona przez Google.

Formularze Google

Influence of climate changes, depression and anxiety on willingness to have a child in Poland

We kindly invite you to complete the survey aimed at finding out which factors influence people's willingness to have or not to have a child in context of climate change, anxiety, stress and depression.

It is a voluntary, anonymous survey carried out by researchers from Medical University of Lodz. The survey consists of three parts — the first one containing general information, the second concerning willingness to have a child and the third assessing severity of stress, anxiety and depression (the standard DASS-21 questionnaire). The condition necessary to take part in the survey is being age over 18. Completing the survey is equal to agreeing to take part in the survey and further data processing gathered in the course of the research. The agreement to take part in the survey can be withdrawn at any time of its duration. The completion of the survey should take you up to ten minutes. The survey received a positive opinion from Bioethical Commission of Medical University of Lodz, decision number RNN/289/21/KE.

Try to complete the survey as thoroughly and honest as you can.

*Wymagane

Basic information

In this section we would like to know you better - you will find here basic questions about your gender, age, place of living and family, which will allow us to characterize respondents group.

1. Sex *

Zaznacz tylko jedną odpowiedź.

- ( ) Male
- ( ) Female
- ( ) Inne: ____________________

2. Age (put a number) *

____

3. Do you live in Poland? *

Zaznacz tylko jedną odpowiedź.

- Yes, I do.
- No, I live abroad.

4. Have you ever lived outside Poland for more than 6 months? *

Zaznacz tylko jedną odpowiedź.

- Yes
- No

5. What is the population of your place of working/studying/spending most of your time? *

Zaznacz tylko jedną odpowiedź.

- City with more than 500 000 residents
- City with more than 250 000 residents
- City with more than 100 000 residents
- City with more than 50 000 residents
- City with less than 50 000 residents
- It is a village

6. What is the population of the place where you were raised? *

Zaznacz tylko jedną odpowiedź.

- City with more than 500 000 residents
- City with more than 250 000 residents
- City with more than 100 000 residents
- City with more than 50 000 residents
- City with less than 50 000 residents
- It is a village

7. What is your formal education? *

Zaznacz tylko jedną odpowiedź.

- Primary education
- Professional training
- Incomplete secondary education
- Complete secondary education
- Higher education

8. Are you still studying? *

Zaznacz tylko jedną odpowiedź.

- Yes
- No

9. Do you have a regular job? *

Zaznacz tylko jedną odpowiedź.

- Yes
- No
- No, I have pension/I am on a sick leave/ I am retired
- I am still studying

10. What is the average monthly net (after deducting costs, allowances and taxes) household income per person in your family? *

Zaznacz tylko jedną odpowiedź.

- less than 1250 PLN
- 1 250 - 2 250 PLN
- 2 250 - 4 250 PLN
- 4 250 - 6 250 PLN
- more than 6250 PLN

11. Are you in a stable relationship (it lasts more than 6 months or is formal)? *

Zaznacz tylko jedną odpowiedź.

- Yes
- No

12. If you are in a stable relationship, for how many years does it last? (if not, please write "n/a"; if lasts less than one year, please write "<1") *

____

13. If you are in a stable relationship, is it formal? *

Zaznacz tylko jedną odpowiedź.

- Yes
- No
- I am not in a steady relationship

14. If you are in an informal relationship, are you planning to formalize it? *

Zaznacz tylko jedną odpowiedź.

- Yes
- No
- I prefer not to answer that question
- I am not in a stable relationship
- Not applicable, I am in a formal relationship

15. Do you belong to any faith/religion? *

Zaznacz tylko jedną odpowiedź.

- Yes
- Formally I do belong, but I do not identify
- No
- I prefer not to answer that question

16. Do you have siblings? *

Zaznacz tylko jedną odpowiedź.

- Yes, biological
- Yes, in-law (one biological parent)
- Yes, adopted
- Yes, mixed (adopted, in-law, biological)
- No

17. Do you keep contact with your family (parents, siblings, grandparents) or part of it? *

Zaznacz tylko jedną odpowiedź.

- Yes
- Yes, but we meet sporadically
- No I do not

18. Your relationship with parents during your childhood, you may describe as: *

Zaznacz tylko jedną odpowiedź.

- Very good
- Good
- Mediocre
- Bad
- Very bad
- I did not have a relationship with them

19. Your relationship with siblings during your childhood, you may describe as: *

Zaznacz tylko jedną odpowiedź.

- Very good
- Good
- Mediocre
- Bad
- Very bad
- I did not have a relationship with them

20. Your relationship with parents in the adulthood you may describe as: *

Zaznacz tylko jedną odpowiedź.

- Very good
- Good
- Mediocre
- Bad
- Very bad
- I do not have a relationship with them

21. Your relationship with siblings in the adulthood you may describe as: *

Zaznacz tylko jedną odpowiedź.

- Very good
- Good
- Mediocre
- Bad
- Very bad
- I do not have a relationship with them

22. I would describe myself as: *

Zaznacz tylko jedną odpowiedź.

- an optimist
- rather an optimist
- a realist
- rather a pessimist
- a pessimist

23. Have you ever been diagnosed with depressive or anxiety disorder? *

Zaznacz tylko jedną odpowiedź.

- Yes, depressive disorder (including bipolar affective)
- Yes, anxiety disorder
- Yes, both depressive and anxiety disorder
- No

Assessment of attitude towards having children

This part applies to your attitude towards having or not having children and describes potential fears and beliefs.

24. How many children do you have? (put the number, if none please write 0) *

____

25. Are they your biological children? *

Zaznacz tylko jedną odpowiedź.

- Yes, they are my biological children
- No, they are adopted/in foster care
- Some of them are biological, some adopted/in foster care
- I do not have children

26. Do you plan to have biological children/decide to have another one? *

Zaznacz tylko jedną odpowiedź.

- Yes, for sure I plan
- Yes, I rather plan
- No, I rather do not plan
- No, for sure I do not plan

27. How many years from now do you want to have your biological child? (if you do not want/you do not know please write "n/a"; if less than one year, put <1) *

____

28. Do you consider adopting a child? *

Zaznacz tylko jedną odpowiedź.

- Yes
- No
- I do not know

29. Does you relationship suffer from infertility? (Infertility is defined as not being able to conceive after 12 months of unprotected sex, at least 3-4 times a week) *

Zaznacz tylko jedną odpowiedź.

- Yes
- No
- I do not know
- Not applicable

In this part of our survey, we present factors that may potentially discourage to have children. Please rate how much it applies to you.

1 - does not discourage at all
2 - rather does not discourage
3 - I have no opinion
4 - rather discourages
5 - definitely discourages

30. Dissatisfying financial condition. *

Zaznacz tylko jedną odpowiedź.

	1	2	3	4	5	
does not discourage at all						definitely discourages

31. Fear of child's congenital defects/diseases. *

Zaznacz tylko jedną odpowiedź.

	1	2	3	4	5	
does not discourage at all						definitely discourages

32. Dissatisfaction with the medical services (e.g. access to gynecologists, obstetricians, pediatricians and prenatal diagnosis). *

Zaznacz tylko jedną odpowiedź.

	1	2	3	4	5	
does not discourage at all						definitely discourages

33. Fear that climate changes will force the offspring to live on the destroyed planet (e.g. increased global average temperature, rising seas levels, extreme weather, resources conflicts). *

Zaznacz tylko jedną odpowiedź.

	1	2	3	4	5	
does not discourage at all						definitely discourages

34. Lack of partner worth having children with. *

Zaznacz tylko jedną odpowiedź.

	1	2	3	4	5	
does not discourage at all						definitely discourages

35. Fear of pregnancy and postpartum complications. *

Zaznacz tylko jedną odpowiedź.

	1	2	3	4	5	
does not discourage at all	◯	◯	◯	◯	◯	definitely discourages

36. Fear of exacerbation of maternal chronic diseases. *

Zaznacz tylko jedną odpowiedź.

	1	2	3	4	5	
does not discourage at all	◯	◯	◯	◯	◯	definitely discourages

37. Responsibilities associated with having a child. *

Zaznacz tylko jedną odpowiedź.

	1	2	3	4	5	
does not discourage at all	◯	◯	◯	◯	◯	definitely discourages

38. Fear associated with professional duties and career. *

Zaznacz tylko jedną odpowiedź.

	1	2	3	4	5	
does not discourage at all	◯	◯	◯	◯	◯	definitely discourages

39. Fear of not being ready to have children. *

Zaznacz tylko jedną odpowiedź.

	1	2	3	4	5	
does not discourage at all	⬭	⬭	⬭	⬭	⬭	definitely discourages

40. Other features that discourage me to have children: (if not applicable, please write "none") *

____

____

____

____

____

In this part of our survey we present factors that may potentially encourage to have children. Please rate how much it applies to you.

1 - does not encourage at all
2 - rather does not encourage
3 - I have no opinion
4 - rather encourages
5 - definitely encourages

41. Increased social services support for children caregivers. *

Zaznacz tylko jedną odpowiedź.

	1	2	3	4	5	
does not encourage at all	⬭	⬭	⬭	⬭	⬭	definitely encourages

42. Lower taxes for children caregivers. *

Zaznacz tylko jedną odpowiedź.

	1	2	3	4	5	
does not encourage at all	◯	◯	◯	◯	◯	definitely encourages

43. Higher income. *

Zaznacz tylko jedną odpowiedź.

	1	2	3	4	5	
does not encourage at all	◯	◯	◯	◯	◯	definitely encourages

44. Better climate policy and decreasing risk of climate crisis. *

Zaznacz tylko jedną odpowiedź.

	1	2	3	4	5	
does not encourage at all	◯	◯	◯	◯	◯	definitely encourages

45. Better healthcare access (obstetricians, gynecologists, pediatricians, prenatal diagnostics). *

Zaznacz tylko jedną odpowiedź.

	1	2	3	4	5	
does not encourage at all	◯	◯	◯	◯	◯	definitely encourages

46. Changes in law giving more privileges for people deciding to have a child. *

Zaznacz tylko jedną odpowiedź.

	1	2	3	4	5	
does not encourage at all						definitely encourages

47. Liberalization of abortion laws. *

Zaznacz tylko jedną odpowiedź.

	1	2	3	4	5	
does not encourage at all						definitely encourages

48. Meeting the right partner. *

Zaznacz tylko jedną odpowiedź.

	1	2	3	4	5	
does not encourage at all						definitely encourages

49. Longer/better paid parental leave. *

Zaznacz tylko jedną odpowiedź.

	1	2	3	4	5	
does not encourage at all						definitely encourages

50. Other features that may encourage me to have children: (if not applicable, please write "none") *

51. Have you or anyone in your family given birth to a child with congenital defects? *

Zaznacz tylko jedną odpowiedź.

- Yes
- No

52. Choose the statements that reflects the best your attitude towards expected climate change, such as increasing average temperature on Earth and rising seas levels: *

Zaznacz wszystkie właściwe odpowiedzi.

- [ ] I am really worried about those issues, I fear the climate crisis
- [ ] I rather care about it
- [ ] I care about it a bit.
- [ ] I do not care about it at all.
- [ ] I try not to think about it.
- [ ] Those expectations are based on wrong assumptions.

53. Do you think that environmental causes (increasing average temperature on Earth, extreme weather, rising seas levels, climate migration) are important reasons for not deciding to have children? *

Zaznacz tylko jedną odpowiedź.

- Yes
- No

54. Do you think that environmental causes present exclusively sufficient reason to decide to not have a child? *

Zaznacz tylko jedną odpowiedź.

- Yes
- No

In the following questions try to describe how much do you agree with the presented statements.

1 - I strongly disagree
2 - I rather disagree
3 - I neither agree nor disagree
4 - I rather agree
5 - I strongly agree

55. Humans cause so much harm – to other humans, animals, and the environment – that it is wrong to procreate. *

Zaznacz tylko jedną odpowiedź.

	1	2	3	4	5	
I strongly disagree						I strongly agree

56. Pregnancy is such an amazing and enriching time in women's life that every woman should experience it at least once. *

Zaznacz tylko jedną odpowiedź.

	1	2	3	4	5	
I strongly disagree						I strongly agree

57. Prospective parents who believe, that their child will have a miserable life should not procreate. *

Zaznacz tylko jedną odpowiedź.

	1	2	3	4	5	
I strongly disagree	⬭	⬭	⬭	⬭	⬭	I strongly agree

58. Having a child equals successful life. *

Zaznacz tylko jedną odpowiedź.

	1	2	3	4	5	
I strongly disagree	⬭	⬭	⬭	⬭	⬭	I strongly agree

59. It is better not to exist than to exist, because only in existence can there be pain and suffering. If one does not exist, one cannot suffer. *

Zaznacz tylko jedną odpowiedź.

	1	2	3	4	5	
I strongly disagree	⬭	⬭	⬭	⬭	⬭	I strongly agree

60. A child is the best evidence of a happy and full of love relationship. *

Zaznacz tylko jedną odpowiedź.

	1	2	3	4	5	
I strongly disagree	⬭	⬭	⬭	⬭	⬭	I strongly agree

61. I fear the climate disaster and the environmental condidtions that my kids will have to/may live in. *

Zaznacz tylko jedną odpowiedź.

	1	2	3	4	5	
I strongly disagree	◯	◯	◯	◯	◯	I strongly agree

62. People deciding to bear children while facing the climate change are irresponsible. *

Zaznacz tylko jedną odpowiedź.

	1	2	3	4	5	
I strongly disagree	◯	◯	◯	◯	◯	I strongly agree

63. I prefer to focus on my personal fulfillment and career rather than to have a child. *

Zaznacz tylko jedną odpowiedź.

	1	2	3	4	5	
I strongly disagree	◯	◯	◯	◯	◯	I strongly agree

64. The Earth is overpopulated, restricted resources do not allow us to reproduce uncontrollably. *

Zaznacz tylko jedną odpowiedź.

	1	2	3	4	5	
I strongly disagree	◯	◯	◯	◯	◯	I strongly agree

65. Deciding not to have children is just a way to avoid taking responsibility. *

Zaznacz tylko jedną odpowiedź.

	1	2	3	4	5	
I strongly disagree	◯	◯	◯	◯	◯	I strongly disagree

66. I am afraid that if I decided to have children they would witness terrible consequences of climate changes. *

Zaznacz tylko jedną odpowiedź.

	1	2	3	4	5	
I strongly disagree	◯	◯	◯	◯	◯	I strongly agree

67. I have always wanted to become a parent. *

Zaznacz tylko jedną odpowiedź.

	1	2	3	4	5	
I strongly disagree	◯	◯	◯	◯	◯	I strongly agree

Ocena nasilenia stresu, lęku i niepokoju/ Assessing of the depression, anxiety and stress levels

In this part we would like to take a look at the intensity of the thoughts and worries related to mood disturbances.
Those questions comes from DASS-21 questionnaire - standardized Depression, Anxiety and Stress Scale. Please, read each question and choose the answer that suggest severity of the symptom in your case within last week.
We suggest to try to be as honest and consistent as it is possible.

The rating scale is as follows and concerns last week:
0 - Did not apply to me at all
1 - Applied to me to some degree, or some of the time
2 - Applied to me to a considerable degree or a good part of time
3 - Applied to me very much or most of the time

68. 1. I found it hard to wind down. *

Zaznacz tylko jedną odpowiedź.

0	1	2	3

69. 2. I was aware of dryness of my mouth. *

Zaznacz tylko jedną odpowiedź.

0	1	2	3

70. 3. I couldn't seem to experience any positive feeling at all. *

Zaznacz tylko jedną odpowiedź.

0	1	2	3

71. 4. I experienced breathing difficulty (e.g. excessively rapid breathing,breathlessness in the absence of physical exertion) *

Zaznacz tylko jedną odpowiedź.

0	1	2	3

72. 5. I found it difficult to work up the initiative to do things *

Zaznacz tylko jedną odpowiedź.

0	1	2	3

73. 6. I tended to over-react to situations. *

Zaznacz tylko jedną odpowiedź.

0	1	2	3

74. 7. I experienced trembling (e.g. in the hands). *

Zaznacz tylko jedną odpowiedź.

0	1	2	3

75. 8. I felt that I was using a lot of nervous energy. *

Zaznacz tylko jedną odpowiedź.

0	1	2	3

76. 9. I was worried about situations in which I might panic and make a fool of myself. *

Zaznacz tylko jedną odpowiedź.

0	1	2	3

77. 10. I felt that I had nothing to look forward to. *

Zaznacz tylko jedną odpowiedź.

0	1	2	3

78. 11. I found myself getting agitated. *

Zaznacz tylko jedną odpowiedź.

0	1	2	3

79. 12. I found it difficult to relax. *

Zaznacz tylko jedną odpowiedź.

0	1	2	3

80. 13. I felt down-hearted and blue. *

Zaznacz tylko jedną odpowiedź.

0	1	2	3

81. 14. I was intolerant of anything that kept me from getting on with what I was doing. *

Zaznacz tylko jedną odpowiedź.

0	1	2	3

82. 15. I felt I was close to panic. *

Zaznacz tylko jedną odpowiedź.

0	1	2	3

83. 16. I was unable to become enthusiastic about anything. *

Zaznacz tylko jedną odpowiedź.

0	1	2	3

84. 17. I felt I wasn’t worth much as a person. *

Zaznacz tylko jedną odpowiedź.

0	1	2	3
⬭	⬭	⬭	⬭

85. 18. I felt that I was rather touchy. *

Zaznacz tylko jedną odpowiedź.

0	1	2	3
⬭	⬭	⬭	⬭

86. 19. I was aware of the action of my heart in the absence of physical exertion (e.g. sense of heart rate increase, heart missing a beat). *

Zaznacz tylko jedną odpowiedź.

0	1	2	3
⬭	⬭	⬭	⬭

87. 20. I felt scared without any good reason. *

Zaznacz tylko jedną odpowiedź.

0	1	2	3
⬭	⬭	⬭	⬭

88. 21. I felt that life was meaningless. *

Zaznacz tylko jedną odpowiedź.

	0	1	2	3
	⬭	⬭	⬭	⬭

Ta treść nie została utworzona ani zatwierdzona przez Google.

Formularze Google
