# Supplementary material for: The Causes and Role of Antinatalism in Poland in the Context of Climate Change, Obstetric Care, and Mental Health
Source: Int J Environ Res Public Health. 2022 Oct 20;19(20):13575. doi: 10.3390/ijerph192013575 (PMC9602747; doi:10.3390/ijerph192013575)
Supplement: Supplementary file 1 [file ijerph-19-13575-s001.zip › Supplement S2, Table S1.pdf]

**Table S1.** Criteria for group assignment.

| Group assignment | | |
|---|---|---|
| Group number | Willingness to have offspring | Having offspring |
| **1**<br>n=55 (4.45%) | + | + |
| **2**<br>n=637 (51.38%) | + | - |
| **3**<br>n=76 (6.14%) | - | + |
| **4**<br>n=472 (38.07%) | - | - |

Legend: + presence of certain attitude; - lack of certain attitude
